# Ant-infecting *Ophiocordyceps* genomes reveal a high diversity of potential behavioral manipulation genes and a possible major role for enterotoxins

**DOI:** 10.1038/s41598-017-12863-w

**Published:** 2017-10-02

**Authors:** Charissa de Bekker, Robin A Ohm, Harry C. Evans, Andreas Brachmann, David P. Hughes

**Affiliations:** 1 University of Central Florida, Department of Biology, Orlando, FL 32816 USA; 2 Ludwig Maximilian University, Faculty of Biology, Planegg-Martinsried, 82152 Germany; 3 Ludwig Maximilian University, Institute of Medical Psychology, Munich, 80336 Germany; 4 Utrecht University, Faculty of Science, Utrecht, 3584 CH The Netherlands; 5 CAB International, E-UK, Egham, Surrey, TW20 9TY United Kingdom; 6 Pennsylvania State University, Entomology and Biology Departments, University, Park, PA 16802 USA

## Abstract

Much can be gained from revealing the mechanisms fungal entomopathogens employ. Especially intriguing are fungal parasites that manipulate insect behavior because, presumably, they secrete a wealth of bioactive compounds. To gain more insight into their strategies, we compared the genomes of five ant-infecting *Ophiocordyceps* species from three species complexes. These species were collected across three continents, from five different ant species in which they induce different levels of manipulation. A considerable number of (small) secreted and pathogenicity-related proteins were only found in these ant-manipulating *Ophiocordyceps* species, and not in other ascomycetes. However, few of those proteins were conserved among them, suggesting that several different methods of behavior modification have evolved. This is further supported by a relatively fast evolution of previously reported candidate manipulation genes associated with biting behavior. Moreover, secondary metabolite clusters, activated during biting behavior, appeared conserved within a species complex, but not beyond. The independent co-evolution between these manipulating parasites and their respective hosts might thus have led to rather diverse strategies to alter behavior. Our data indicate that specialized, secreted enterotoxins may play a major role in one of these strategies.

## Introduction

Fungi have evolved to occupy a wide variety of ecologically important niches. Saprophytic fungi are crucial players in the carbon cycle. Mutualistic and parasitic fungi evolved to intimately interact with their plant and animal hosts. A consequence of this broad niche space and the ability of fungi to secrete useful enzymes and bioactive compounds is their successful use in a wide variety of industries (e.g. medicine, food, biopesticides). Much can thus be gained from sequencing fungal genomes and discovering the enzymes and mechanisms they employ. From a medical perspective, this is especially true for fungi that interact with animals. Certain fungi have evolved to interact in mutualistic ways to provide the animal host with beneficial compounds^1,2^. Others can overcome the immune system and take advantage of the host to further transmission^3^. Many such interactions can be found with arthropods since they are the most diverse and dominant animals in all terrestrial ecosystems^4^, and fungal parasites of insects (entomopathogens) can be found in all major phyla^5^. Despite this, fungal-insect associations remain understudied^6^ in comparison to fungal parasites of plants. This imbalance is unfortunate given the many insights that could be gained from determining the mechanisms by which fungi invade and conquer their insect hosts.

The fungal parasites that adaptively manipulate the behavior of their insect hosts are particularly intriguing because such manipulation implies many interesting bioactive compounds are likely produced during infection^7^. Studies into the mechanisms underlying these manipulations may reveal the fungal neuromodulators that are secreted and the pathways that give rise to certain behaviors. Prime examples of fungal manipulation of insect behavior are those where ants are manipulated to leave the nest and bite onto vegetation in elevated positions. Examples of this are wood ants, *Formica*, infected by *Pandora* (Phylum: Entomophthoromycota, Order Entomophthorales)^8–10^, and carpenter ants and spiny ants (tribe Camponotini, the genera *Camponotus* and *Polyrhachis*, respectively) infected by fungi of the genus *Ophiocordyceps* (Phylum: Ascomycota, Order Hypocreales)^11–16^. These *Ophiocordyceps* species can be isolated from their ant hosts and grown *in vitro*. As such their biology can be studied in more detail by looking at the fungal secretome under various circumstances and reconstructing ant infection and manipulation under controlled conditions^17^. The biting behavior for one of the species, *Ophiocordyceps unilateralis sensu lato* (*s.l*.) from South Carolina, USA, has recently also been studied from a whole genome perspective. Further, a mixed transcriptomics study that compared gene expression at this biting event with the situation after manipulation and before ant and fungus were interacting, has revealed the very first candidate genes that could be important in establishing the behaviors observed^18^.

**Figure 1:**
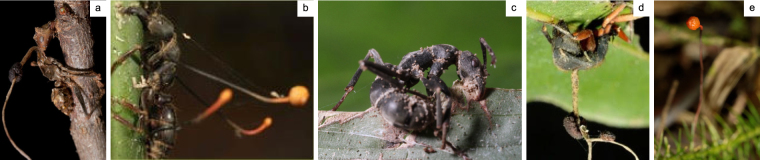
Ant-infecting fungi of the genus *Ophiocordyceps*. Photographs of the five ant-infecting *Ophiocordyceps* fungi compared in this study as they emerge from their hosts. (**a**) *O. kimflemingiae* emerging from a *C. castaneus* ant that has died biting a twig and grasping it with the legs. Ghanaian Ponerine ants infected by local (**b**) *O. australis s.l*. and (**c**) *O. subramanianii s.l*. species that were manipulated to bite on vertical stems of small, understory vegetation. (**d**) *O. camponoti-rufipedis* emerging from a leaf biting *C. rufipes* ant, collected in the Atlantic rainforest of Brazil. (**e**) An *O. australis s.l*. species from that same region as found on Ponerine ants. Here, biting behavior is not induced and the ant dies in the leaf litter.

Entomopathogenic Ascomycota mostly reside within the Order Hypocreales, where multiple origins of the host behavioral manipulation trait can be found^5^. As such, *Camponotus* and *Polyrhachis* are not the only ants infected by *Ophiocordyceps*. Each ant species is seemingly infected by its own *Ophiocordyceps* species, creating a high level of biodiversity among the ant-manipulating *Ophiocordyceps* fungi^19,20^. The description of the vast majority of these species is still underway. In the interim, species complex names are assigned to these *Ophiocordyceps* fungi based on their fruiting body and ascospore morphology, and the ant species they infect. For example, *O. unilateralis s.l*. is found on Camponotini species (*Camponotus* and *Polyrhachis)* and forms a stalk with the fruiting body (ascoma) attached to one side (hence the epithet *unilateralis*). Spores are long and more or less “boomerang shaped”^13^. *Ophiocordyceps australis s.l*. is found on Ponerine ants, where the fruiting body at the tip of the stalk produces chains of shorter (part-) spores that are released in a fragmented manner^5,20^. Here, we improved the assembly and annotation of a previously sequenced^18^ ant-manipulating *Ophiocordyceps* species, *Ophiocordyceps kimflemingiae* ^20^, a *unilateralis* species from the United States. Additionally, we sequenced, assembled, and annotated the genomes of four additional species: *Ophiocordyceps camponoti-rufipedis* ^13^, a species of the *O. unilateralis* complex from Brazil; two species of the *Ophiocordyceps australis* complex from Brazil and Ghana; and a species from yet another complex, *Ophiocordyceps subramanianii s.l*., also from Ghana^5^. Phylogenetics studies that demonstrate species delimitation within the *O. australis* complex have not yet been published at this time. We, however refer to the two sequenced *australis* strains with “species” as per the one fungus-one ant theory^19,20^. *Ophiocordyceps kimflemingiae* is from a temperate system, where infected *Camponotus castaneus* ants are found biting twigs and grasping them with their legs in what is a novel form of manipulation distinct from biting^17^. Biting was also seen in tropical Ghanaian Ponerine ants infected by local *O. australis s.l*. and *O. subramanianii s.l*. species. In this case biting is typically on vertical stems of small, understory vegetation in tropical forests^19^. The fungus *O. camponoti-rufipedis*, in the tropical Atlantic rainforests of Brazil, induces leaf biting in its *Camponotus rufipes* hosts^13^, whereas the *O. australis s.l*. species on Ponerine ants from that region does not induce biting behavior^20^ (Fig. 1). For the *O. australis* species from Brazil, we thus cannot say with certainty if manipulation is taking place (because the biting event is absent), while the other species induce evident manipulated behaviors (the biting event is present).

According to various “-omics” studies, parasitic traits, such as host invasion and overcoming the immune system, rely on molecular strategies that share many commonalities across plant and animal parasite species^21^. One would thus expect to observe something similar when investigating possible strategies that fungal parasites use to manipulate the behavior of their animal hosts. Especially if all hosts compared reside within the same family, the Formicidae, and all parasites within the same genus, *Ophiocordyceps*. Through comparative genomics, we investigated the possible common mechanisms that these fungi employ to manipulate the behavior of their individual ant hosts. In addition, we compared their genomes to several other entomopathogenic and non-entomopathogenic ascomycetes in order to ascertain if genes related to pathogenicity and manipulation are also found in other species or are specific to this group. Moreover, we asked how conserved the identified candidate manipulation genes are by re-analyzing the previously published transcriptome of *O. kimflemingiae* ^18^. We then aimed to infer which genes might be good first candidates to focus on in follow-up studies to quantitatively assess their true function. This led us to conclude that a deeper understanding of the function of fungal genes encoding for putative secreted enterotoxins (i.e. fungal genes that have an enterotoxin_a PFAM domain as well as a secretion signal) should be obtained.

## Results

### Genome features

The *Ophiocordyceps* draft genomes generated in this study (Table 1) were assembled with reads generated across various sequencing runs using two types of DNA libraries. Only the runs that led to high-quality reads were used for assembly (see Materials and Methods). Contig assembly resulted in genome sizes ranging from 21.91–23.92 million base pairs (Mbp) for *O. unilateralis s.l*. and *O. australis s.l*. species. In contrast, *O. subramanianii s.l*. had an estimated genome size of 32.31 Mb. Gene prediction yielded between 7,621 and 8,629 genes for the *O. unilateralis* and *O. australis* species. *Ophiocordyceps subramanianii s.l*. was predicted to have 11,275 genes. Additionally, GC content in *O. subramanianii s.l*. (i.e. 60.35%) was much higher compared to the other *Ophiocordyceps* species in this study (54.66%+/− 1.57%) (Table 1).

**Table 1:** Features of *Ophiocordyceps* draft genomes.

Property	*O. kimflemingiae* – USA (SC16a)	*O. camponoti-rufipedis* – Brazil (Map-16)	*O. australis sensu lato* – Brazil (Map-64)	*O. australis sensu lato* - Ghana (1348a)	*O. subramanianii sensu lato* – Ghana (1346)
Fold coverage	130	29	135	20	19
Scaffolds in assembly	2537	2206	594	2297	3401
Total assembly length (Mbp)	23.92	21.91	23.32	22.19	32.31
N50 (kb)	27	22	112	17	17
Largest scaffold (kb)	167	147	428	118	139
Assembly GC content (%)	55.92	56.1	53.13	53.48	60.35
Transcript GC content (%)	59.84	60.57	56.74	56.98	64.43
Assembly gaps (%)	0.75	0.01	0.4	0.55	0.38
Repetitive content (%)	6.83	6.59	2.87	2.45	4.06
Genes	8629	7621	8174	7995	11275
Gene length (median)	1294	1350	1363	1313	1039
Transcript length (median)	1152	1200	1227	1182	900
Exon length (median)	220	273	268	290	266
CDS length (median)	1149	1200	1227	1182	900
Protein length (median)	383	400	409	394	300
Spliced genes (total, %)	6871 (79.63%)	5777 (75.8%)	6258 (76.56%)	5952 (74.45%)	7705 (68.34%)
Exons per gene (median)	3	2	2	2	2
Intron length (median)	62	60	64	65	71
Introns per spliced gene (median)	2	2	2	2	2
Gene density (genes/Mbp)	360.69	347.89	350.45	360.3	348.92
Unique PFAM domains	3499	3396	3542	3482	3446
Genes with PFAM (total, %)	5749 (66.62%)	5359 (70.32%)	5949 (72.78%)	5746 (71.87%)	6379 (56.58%)
Genes with GO (total, %)	3981 (46.14%)	3765 (49.4%)	4143 (50.69%)	4000 (50.03%)	4387 (38.91%)
Genes with signalP (total, %)	914 (10.59%)	840 (11.02%)	802 (9.81%)	681 (8.52%)	1064 (9.44%)
Genes with TMHMM (total, %)	1536 (17.8%)	1391 (18.25%)	1537 (18.8%)	1469 (18.37%)	1752 (15.54%)
CEGMA completeness (%)	99.13	98.69	99.13	98.25	98.47
BUSCO complete or fragmented (%)	97.6	93.6	98.2	95.9	89.6

We also improved the assembly and gene prediction of the previously published genome of *O. unilateralis s.l*. strain SC16a (Supplementary Table S1), which has now received the species name *O. kimflemingiae* ^20^. In addition to being less fragmented, the new assembly is smaller than previously reported^18^. This is most likely due to a better assembly of repetitive regions, which were found highest in the *unilateralis* species (6.59–6.83%) compared to the other-ant-infecting species in this study (Table 1). Despite the smaller assembly size, the new gene prediction increased the gene count with 798 genes. This increase is predominantly caused by the prediction of fewer chimeras (i.e. neighboring genes that are incorrectly merged into one gene model) using the Braker1 pipeline (data not shown).

For all but one of the genomes generated in this study, gene prediction was informed by RNA-Seq reads. For the previously reported North American *unilateralis* species *O. kimflemingiae*, reads generated in that former study were used^18^. Through difficulties with cultivating Brazilian *O. camponoti-rufipedis*, not enough material was obtained to generate RNA-Seq data in addition to the DNA reads needed to obtain a draft genome. Because both *O. kimflemingiae* and *O. camponoti-rufipedis* reside within the same species complex (*unilateralis)*, we attempted mapping the North American *unilateralis* species reads to the *O. camponoti-rufipedis* genome to see if they could be used to inform annotation. However, while 93% of the *O. kimflemingiae* reads mapped to its own genome, only 43% mapped to the *O. camponoti-rufipedis* genome (Supplementary Table S2). We additionally mapped the reads to another published *O. unilateralis s.l*. genome, that of *O. polyrhachis-furcata* ^22^, to determine whether this would be a more general cross-mapping effect or specific to the *O. camponoti-rufipedis* genome. A similar mapping of 41% resulted. This suggests that *unilateralis* species might generally be rather distantly related, making cross-mapping to inform annotation less suitable. For informative purposes, we also cross-mapped the *australis* species with each other (strains MAP-64 from Brazil and 1348a from Ghana). This resulted in 71% and 82% cross-mapped reads versus 86% and 97% mapped to their own genomes, respectively (Supplementary Table S2). This implies that species in the *australis* complex are thus likely much more closely related to each other than species within the *unilateralis* complex.

### Orthologous clusters

**Figure 2:**
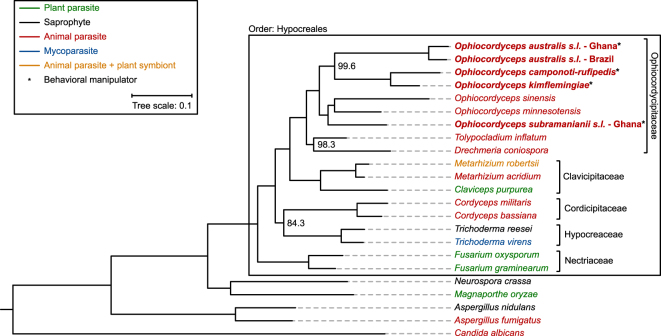
Phylogenetic relationship of ant-infecting *Ophiocordyceps* species with other sequenced and annotated ascomycetous fungi. Fungal lifestyles are indicated with different colors. Genomes generated in this study are indicated in bold. Unless indicated otherwise, bootstrap values were 100.

We set out to investigate which gene predictions of the ant-infecting *Ophiocordyceps* species in this study are likely conserved and shared with other ascomycetes. In addition, we aimed to discover what species-specific and “manipulation-specific” specializations might have taken place. As such the predicted proteomes of our five ant-infecting fungi were compared to those of 18 other ascomycetous fungi. Of these species, ten were animal parasites (two infecting mammals, two infecting nematodes, and six infecting insects) and 13 reside within the same order (Hypocreales). Four species belonged to the same family (Ophiocordycipitaceae) and two were within the same genus (*Ophiocordyceps*). A phylogenetic reconstruction based on 67 conserved genes present in each of these organisms is depicted in Fig. 2.

**Figure 3:**
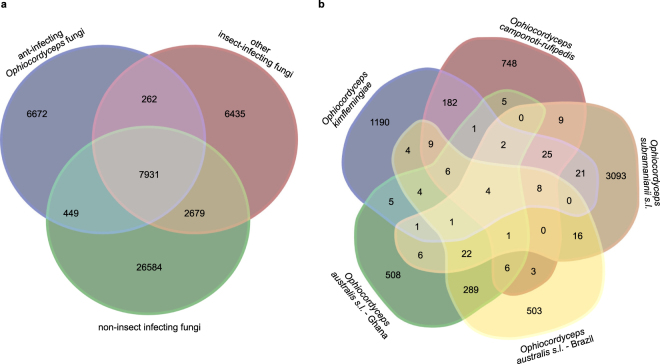
Venn diagrams of orthologous clusters. (**a**) Venn-diagram of orthologous clusters present within three fungal species ranges: ant-infecting entomopathogens of the genus *Ophiocordyceps* (blue), other entomopathogens (red), and other ascomycetes in this study (green, ranging from animal-, fungus- and plant-infecting, to saprophytic fungi). (**b**) Venn-diagram of the 6672 orthologous clusters found only in ant-infecting species, specified into the five *Ophiocordyceps* species that comprised this species range. A Venn-diagram of all orthologous clusters found in ant-manipulating species is depicted in Supplementary Figure S2.

A total of 51,012 orthologous clusters included all annotated proteins of each of the 23 ascomycetes included in this analysis (Supplementary Data S1). Summary statistics regarding this clustering can be found in Supplementary Figure S1. We compared overlap of orthologous clusters between three species ranges: 1) the ant-infecting fungi, which comprised all five draft genomes generated in this study, 2) the other insect-infecting fungi *Ophiocordyceps sinensis*, *Tolypocladium inflatum*, both *Metarhizium* and both *Cordyceps* species, and 3) all non-insect-infecting ascomycetes, which included other animal-infecting, plant-infecting, fungus-infecting, and saprophytic fungi. The results of this analysis are depicted in the Venn Diagram of Fig. 3a. Subsequently, we performed enrichment analyses for the functional annotations of ant-infecting species genes that were found within the various overlapping and non-overlapping parts of the diagram. Of the 7,931 orthologous clusters that were found within all three species ranges, Gene Ontology (GO) annotations for general biological processes were significantly over-represented. This suggests, as one would expect, that ascomycetes with different lifestyles use similar mechanisms for general processes such as transcription, translation, protein transport and signal transduction. However, genes predicted to encode for (small) secreted proteins, proteins with GO annotations for multi-organism processes and pathogenesis, and more specifically, secreted putative enterotoxins, were under-represented. Indeed, when we performed an enrichment analysis of annotations present in orthologous clusters that were only found in the ant-infecting species (i.e. 6,672 clusters, Fig. 3a), we found the opposite result. GO annotations for (largely the same) general biological processes were significantly under-represented, while (small) secreted proteins, proteins with GO annotations for multi-organism processes and pathogenesis, and putative enterotoxins were over-represented. This suggests that a significant part of the secretome of the ant-infecting fungi in this study is specific to them. This specificity appeared partly due to enterotoxins that are part of the secretome and bioactive small secreted proteins (SSPs) that could be important in fungal-ant interactions. (Small) secreted proteins were also over-represented among the clusters that ant-infecting fungi exclusively shared either with other entomopathogens (262 clusters) or non-entomopathogens (449 clusters). This indicates that their secretomes also contain more general entomopathogen-specific proteins, as well as proteins that are exclusively shared with non-entomopathogenic ascomycetes.

We also examined how orthologous clusters, that were only found among the ant-infecting fungi were represented by those species (Fig. 3b). Of the total of 6,672 clusters in this comparison, 90.6% appeared to be species-specific. Cluster overlap was thus marginal even though all species in this study reside within the same genus (*Ophiocordyceps*) and in some cases even within the same species complex (*O. unilateralis s.l*. and *O. australis s.l*.). Analyzing enrichment of functional annotation terms in these species-specific clusters again resulted in over-representations of (small) secreted proteins. Though marginal, most cluster overlap was found between more closely related species within the same complex (i.e. *O. australis s.l*., 289 clusters, and *O. unilateralis s.l*., 182 clusters Fig. 3b). Also here, enrichment analyses revealed an over-representation for (small) secreted proteins. Thus, it appears that a statistically significant amount of the fungal secretome of these ant-infecting species is either complex- or species-specific. In addition, we found an over-representation of pathogenicity GO terms among the complex-specific orthologous clusters. This finding could be attributed to the presence of orthologous enterotoxins in species within the same complex. Only four clusters that were not present in any of the other ascomycetes in our comparison were shared among all five ant-infecting species (Fig. 3b). None of these four clusters received a functional annotation, but three of them contained genes with predicted secretion signals. For three out of the four clusters, a BLASTp analysis of the genes against the NCBI database resulted solely in hypothetical protein hits with the previously deposited version of the *O. kimflemingiae* genome^18^. This indicates that these clusters might indeed represent proteins that are unique to ant-infecting *Ophiocordyceps* species. Protein sequences within the fourth cluster resulted in hits with metalloproteases in addition to aligning again with a hypothetical protein from *O. kimflemingiae* (XA68_3159), (Supplementary Table S3). This cluster might thus contain putative metalloproteases that are solely found in the genomes of the ant-infecting fungal species examined here. Moreover, only 2 of the clusters found uniquely in the ant-infecting fungi were shared among all four species that induce biting behavior (Fig. 3b). A BLASTp analysis of the genes within these clusters again resulted in hypothetical protein hits with the previously deposited version of the *O. kimflemingiae* genome^18^ (Supplementary Table S4).

### Candidate manipulation genes involved in establishing biting behavior

We analyzed the conservation of candidate genes associated with the manipulated biting event observed in infected ants. We used previously published transcriptomics data^18^ and re-determined differential gene expression by mapping the data to the new version of the *O. kimflemingiae* genome. As per the previously published study, we followed the reasoning that candidate genes, involved in establishing manipulated biting behavior, would be up-regulated during this event and quickly down-regulated again after. As such, 547 candidate genes were identified, which is 49 more than reported in the previous analysis^18^. In line with the previously reported data, genes involved in DNA replication, oxidation-reduction processes, secretion and secondary metabolism were over-represented.

**Figure 4:**
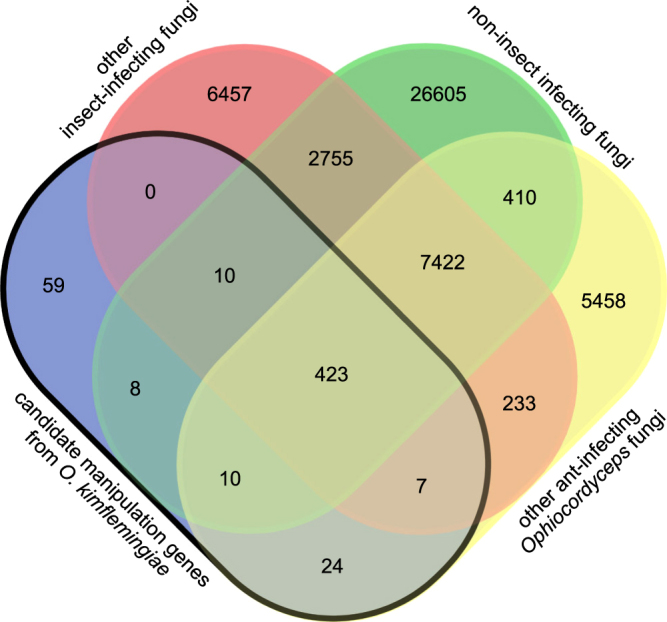
Conservation of candidate manipulation genes. Venn-diagram of orthologous clusters present within the subset of *O. kimflemingiae* candidate manipulation genes, other ant-infecting species, other insect species and other ascomycetes included in this study.

Conservation of candidate manipulation genes was also analyzed by way of orthologous clustering. We compared overlap of orthologous clusters containing those candidate genes, that were significantly up- during and down-regulated after manipulated biting behavior in *O. kimflemingiae*, with three species ranges: 1) the other ant-infecting fungi, which comprised the four new draft genomes generated in this study, 2) the other insect-infecting fungi, and 3) all non-insect-infecting ascomycetes used for comparison earlier. The results of this analysis are depicted in the Venn Diagram of Fig. 4. Of the candidate manipulation genes, 78% appeared to be orthologs of genes present in all other three species ranges (i.e. 423 clusters, Fig. 4). This implies that the genes expressed during manipulated biting induced by *O. kimflemingiae* are not likely specific to manipulation. Among these broadly shared genes, CYPs and other oxidation-reduction-related functions were over-represented, as well as genes encoding for secreted proteins and proteases. Various secondary metabolism annotations (clusters 7,8 and 9) were also over-represented among these more broadly shared orthologous clusters. They comprised a tryptophan dimethylallyltransferase involved in ergot alkaloid synthesis, various cytochromes, small secreted proteins, a polyketide synthase (PKS), and a PKS-NRPS (non-ribosomal protein synthetase) hybrid. Among the candidate manipulation genes that appeared to be unique to *O. kimflemingiae* (i.e. 59 clusters, Fig. 4), only SSPs were over-represented. However, 92% of these “unique” genes did not receive a functional annotation. Where any other overlap with the three species ranges was found, SSPs were also over-represented, as were larger secreted proteins. Of those present only in ant-infecting fungi (i.e. 24 clusters Fig. 4), again the majority (79%) did not receive a functional annotation. Among the seven orthologous clusters that were present in all insect-infecting species, but not in other ascomycetes, we found a putative, secreted enterotoxin. This enterotoxin was present in the biting behavior-inducing species *O. kimflemingiae* (two orthologs), *O. camponoti-rufipedis* (1 ortholog), *O. subramanianii s.l*. (two orthologs) and *O. australis-*Ghana (two orthologs), as well as in *O. australis*-Brazil and *O. sinensis*. Moreover, one of the two enterotoxin orthologs in *O. kimflemingiae* showed a dramatic expression pattern with a >3,000-fold up-regulation during manipulation and a 200-fold down-regulation after^18^. This enterotoxin could thus potentially be an important key player in the establishment of behavioral manipulation by these *Ophiocordyceps* species.

### Conservation of secondary metabolite clusters

**Figure 5:**
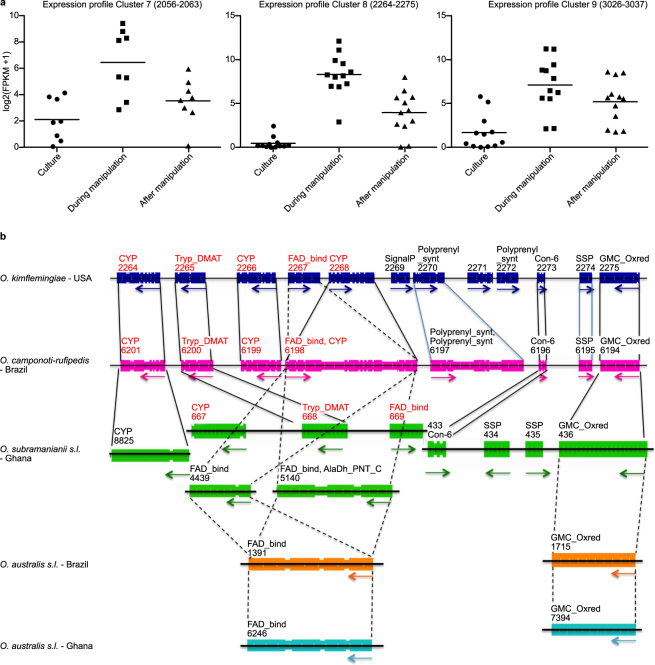
Secondary metabolite clusters in behavior manipulating *Ophiocordyceps* species. (**a**) Expression profiles of three differentially expressed secondary metabolite clusters in *O. kimflemingiae* in culture, during manipulated biting behavior, and after manipulation. (**b**) Homology (identified by BLASTp alignment) and orthology (identified by orthologous clustering) of genes within secondary metabolite cluster 8. Genes that were found using both methods are connected by a black line. Genes found by BLASTp alignment only are connected with a blue line, while genes only found by orthologous clustering are connected with a dashed line. Gene numbers and functions in red indicate genes that received a secondary metabolism annotation.

A general hypothesis is that altered host behaviors are established through the secretion of secondary metabolites in addition to larger bioactive compounds. This hypothesis is supported by the over-representation of certain annotated secondary metabolite clusters among genes of *O. kimflemingiae* that are up-regulated during manipulated biting behavior^18^. Examining these more closely, we found that indeed the genes, within but also directly flanking the annotated secondary metabolite clusters, followed this particular expression pattern (Fig. 5a). We asked if these clusters were conserved among insect-infecting *Ophiocordyceps* fungi. This would suggest a similarity in use of secondary metabolites by these fungi to interact with their ant hosts to establish the observed manipulated behaviors. As such, we examined annotated clusters 7,8 and 9 of *O. kimflemingiae* and their directly flanking genes. These clusters 1) were up-regulated during manipulated biting behavior followed by a significant down-regulation, and 2) shared orthologs with other ascomycetes (see above). For the genes within these clusters, we searched for homologs (BLASTp alignment) and orthologs (orthologous clustering) in the other four ant-infecting fungi (Fig. 5b and Supplementary Figure S3). This demonstrated that the *unilateralis* species organized at least some of their secondary metabolism-related genes in largely similar clusters. The other *Ophiocordyceps* species had homologs and orthologs of these secondary metabolism genes scattered throughout their genomes or did not contain a copy at all (Fig. 5b and Supplementary Figure S3). For instance, annotated cluster 8 in *O. kimflemingiae* contained a tryptophan dimethylallyltransferase flanked by oxidation-reduction-related CYPs and a gene with a FAD binding domain. This cluster is directly flanked by seven genes that followed a similar expression pattern (Fig. 5a). The *O. camponoti-rufipedis* genome had this tryptophan dimethylallyltransferase in a similar fashion; flanked by CYPs and an FAD-binding gene, followed by homologs and orthologs of the neighboring genes (Fig. 5b). The *O. subramanianii s.l*. genome also had a similar tryptophan dimethylallyltransferase. It was however flanked by non-homologous/orthologous CYP and FAD-binding genes. In fact, *O. subramanianii s.l*. did have homologs/orthologs of these genes but they resided on completely different contigs, as did the genes right outside the annotated secondary metabolite cluster (Fig. 5b). Moreover, both *australis* species did not have an orthologous/homologous gene encoding for this particular tryptophan dimethylallyltransferase. In fact, the *australis* species from Ghana appeared to not contain a putative tryptophan dimethylallyltransferase at all. Similar conclusions could be made analyzing the other secondary metabolite clusters (Supplementary Figure S3). Clusters 7 and 9 from *O. kimflemingiae* seemed largely comparable to clusters 31 and 8 from *O. camponoti-rufipedis* respectively. Yet, genes associated with these clusters were again either not present or scattered across the genomes of the three other ant-infecting species.

### Phylogeny of fungal enterotoxins

Genes that contained an annotated enterotoxin PFAM domain (PF01375) and a secretion signal were indicated as putative, secreted enterotoxins. Previous results and our current study implicate that genes encoding for these putative enterotoxins are of importance in behavior-manipulating species of *Ophiocordyceps* fungi. These secreted bacterial-like toxins could potentially be affecting ant behavior by interfering with the production of chemo signaling molecules in the host. This was demonstrated for enterotoxins from bacterial entomopathogens in sex pheromones of boll weevils^23,24^. They could however also function as mere killing compounds^25^. In addition, one of these putative enterotoxins was extremely highly up-regulated during the manipulated biting event only^18^. This particular gene was present in all ant-manipulating *Ophiocordyceps* species in this study, as well as *O. australis*-Brazil and *O. sinensis*. Moreover, the ant-infecting fungi appeared to have a rather large number of genes encoding for these pathogenicity-related proteins. Other ascomycetes generally contained either far fewer (i.e. other entomopathogens, nematode-infecting fungi and *Magnaporthe oryzae*) or no enterotoxin domain-containing genes (i.e. the remainder of the ascomycetes in this study). The genomes of ant-infecting *Ophiocordyceps* species had 20–36 putative enterotoxins with *O. australis-*Ghana (n = 20) having the smallest and *O. kimflemingiae* (n = 36) having the largest number. Nematode-infecting fungi *Ophiocordyceps minnesotensis* and *Drechmeria coniospora* contained 19 and 25 genes with an enterotoxin PFAM domain. Other entomopathogens of the order Hypocreales contained only 4–16 enterotoxin annotations with *Metarhizium robertsii* and *Cordyceps bassiana* having the most (n = 16 and n = 14, respectively). The other ascomycete genomes in this study did not have any enterotoxin-encoding genes, except for the plant pathogen *M. oryzae*, which contained six.

We built a phylogenetic tree based on all fungal genes in this study that contained the PF01375 heat-labile enterotoxin alpha chain domain. Enterotoxins have mostly been reported for bacterial species (e.g. refs ^23–25^). We therefore also included four bacterial enterotoxins. This resulted in a tree based on 252 domain regions. We first determined how the enterotoxins of the bacterial and plant-infecting species (i.e. *M. oryzae*) would cluster with respect to the majority of animal-infecting species (i.e. insect- and nematode-infecting fungi). This placed three bacterial enterotoxins in one clade, and the fourth in a separate clade (Supplementary Figure S4). One bacterial enterotoxin, from *Leptospira mayottensis*, formed an outgroup for the plant-infecting *M. oryzae* clade. *M. oryzae* resides outside the order Hypocreales. As such, the plant pathogenic enterotoxins at of this species were used as an outgroup and the tree was rooted on *L. mayottensis* (Supplementary Figure S4). This tree showed that some enterotoxins of ant-infecting fungi are related to those of other insect- or nematode-infecting species. Other enterotoxins formed their own clades. Within these ant infection-specific clades, enterotoxins from the two *unilateralis* complex species or the *australis* complex species were often paired. *Ophiocordyceps subramanianii s.l*. enterotoxins often formed the outgroup to a *unilateralis-* or *australis-*specific clade (Supplementary Figure S4). In addition, the enterotoxin that was highly up-regulated during manipulated biting behavior (i.e. GeneID Ophio5|373 Supplementary Figure S4), as well as conserved among all ant-infecting species in this study (and *O. sinensis*, see above), resided within a clade that only contained ant-manipulating species. This indicates that this particular enterotoxin could indeed be of key importance in establishing manipulated behavior as observed in the species included in this study.

## Discussion

Much insight about the functioning of a species can be gained from its annotated genome. As such, we sequenced and annotated the genomes of *Ophiocordyceps* fungi that manipulate the behavior of their respective ant hosts. Additionally, we improved the assembly and annotation of a previously published genome. These species were collected across three continents (Africa, North America, and South America), and induced different biting behaviors in their respective ant host species. One of the species did not induce biting behavior, making the presence of manipulation less evident. To gain insight into the mechanisms these parasites employ, we compared their genetic information. The *unilateralis* and *australis* species of this genus had comparable genome features. *Ophiocordyceps subramanianii s.l*. was different in various aspects with the biggest difference being gene number and genome size. Our phylogenetic analysis indicated that *Ophiocordyceps* species within the same species complex (i.e. *unilateralis* and *australis*) form sister taxa. This is according to our expectations since fungi within a species complex are assumed to have closely co-evolved with taxonomically related ant host species (i.e. Camponotini and Ponerines for the *unilateralis* and *australis* clades respectively). In fact, there is growing evidence for the hypothesis that this close co-evolution has led to “one ant-one *Ophiocordyceps* species”^11,13,20,26,27^. Our read mapping efforts suggested that species within the *australis* complex are more closely related to each other than those residing within the *unilateralis* complex, even though one of them does not induce manipulated biting while the other *australis* does. Indeed, RNA-Seq reads from one *unilateralis* species could not be used to inform the annotation of another species within that complex due to low percentage mapping. Related to this is the fact that the two species in question (*O. kimflemingiae* and *O. camponoti-rufipedis*) come from separate continents (North vs. South America, respectively) and likely have very distinct biogeographical origins. In contrast, cross-mapping between *australis* species resulted in high percentage mapping. This again might be related to biogeography as South America and Africa were joined and their biota overlaps considerably^28^. The taxonomic broadness of the ant hosts that the *unilateralis* species infect might also explain their decreased relatedness. To illustrate, the tribe Camponotini consists of eight genera with the genus *Camponotus* containing 46 subgenera and over 900 species^29^. As such, the ant hosts infected by the species used in this study are part of different subgenera (*Tanaemyrmex* and *Myrmothrix* ^30^). The seemingly decreased genomic relatedness of these *unilateralis* species could thus be a result of a continued close co-evolution with their respective hosts.

Orthologous clustering demonstrated that the ant-infecting fungi sequenced in this study share general processes with other ascomycetes. These processes include transcription, translation, protein transport and signal transduction. However, a significant number of signal peptide-containing genes were not shared. These secreted proteins comprised a significant number of small secreted, potentially bioactive, proteins, and enterotoxins. These enterotoxins have largely been reported as bacterial toxins^23–25^, and have previously only been mentioned for a hand full of fungal species regarding their presence in the genome but without a discussed function^18,22,31–34^. The vast majority of the orthologous clusters that were specific for the five ant-manipulating species were also not shared across them. In fact, a significant part of the secretome appeared specific for each individual *Ophiocordyceps* species. Yet, the majority of the candidate manipulation genes, identified through RNA-Seq, were not unique to *O. kimflemingiae* nor to ant-infecting *Ophiocordyceps* species. They generally shared orthologs with at least one other ascomycete in this study. This suggests that the genes up-regulated during manipulated biting behavior in *O. kimflemingiae*, are largely not completely unique to that species. This could mean that these fungi do not use an exclusive set of novel genes to establish the respective complex behaviors observed. Instead, a variety of combinations of genes that are also found in other ascomycetes could be employed. The fungi sequenced in this study were collected on different continents where they induced different (levels of) manipulations (e.g. biting is present or absent) in different ant species. Their strategies at the genetic level could thus be very different as well. Alternatively, sampling during established biting behavior might not give us the correct pool of candidate manipulation genes. We found that a significant number of putatively secreted proteins are unique to the ant-manipulating fungi in this study. These proteins are however not significantly represented in the analyses regarding the candidate manipulation genes. It could be that we are missing out on many of the true candidates when we are sampling at the time when biting is already established. A better insight may be obtained by sampling during the time leading up to this event. This however demands that the suite of subtler behavioral changes leading up to the ultimate biting event is better quantified first.

We also looked more specifically at the conservation of secondary metabolite clusters that could play a role in the manipulation of ant behavior. Indeed, a variety of them are significantly up-regulated during manipulated biting behavior in infected *C. castaneus* ants^18^. We looked at three of these clusters from *O. kimflemingiae* in more detail, of which one contained a putative tryptophan dimethylallyltransferase. This enzyme facilitates the first step in the ergot alkaloid biosynthesis pathway^35^. Our analysis showed that secondary metabolite clusters that are activated during manipulated biting behavior are not conserved across *Ophiocordyceps* species complexes. They do however seem to be largely conserved within a species complex. Seemingly, secondary metabolite clusters are largely shared among *unilateralis* species that infect Camponotini. So, while these species seem rather distinct regarding their genes that encode for the suite of secreted enzymes, they might actually employ more similar secondary metabolite pathways. The suite of secondary metabolites involved in parasite-host interactions that lead to behavioral manipulation might thus translate rather well across *Ophiocordyceps* fungi within a species complex.

Ultimately, our efforts pointed towards a possible major role that enterotoxins might play in the parasite-host interactions between *Ophiocordyceps* fungi and their ant hosts. Not all ascomycetous genomes in our study contain secreted enterotoxins though. In contrast, ant-infecting *Ophiocordyceps* fungi contain many more genes in their genomes encoding for these secreted compounds compared to other ascomycetes. A significant proportion of these genes, therefore, appear to be specific to these species. Our orthologous clustering and phylogenetic tree analysis also suggested that fungi that reside within the same complex (*unilateralis* or *australis*) have genetically similar enterotoxins. Previous transcriptomics data of *O. kimflemingiae* suggests that enterotoxins are dynamically up- and down-regulated. Indeed, one of the enterotoxins looks especially interesting as it is extremely highly up-regulated during the manipulated biting event, after which it is immediately significantly down-regulated again^18^. This gene has orthologs across all five ant-infecting *Ophiocordyceps* species in this study. It could thus be a key component in establishing the altered behaviors we observe in infected ants. The function of this fungal enterotoxin is not clear at this time. However, the literature on bacterial enterotoxins in insect hosts indicates that it might impair the host’s chemosensory abilities^23,24^. More studies into fungal enterotoxins are needed, however, as well as functional genetics studies that would truly determine how *Ophiocordyceps* species might use these compounds to interact with their ant hosts.

## Methods

### Strain culturing and maintenance

*Ophiocordyceps camponoti-rufipedis* strain MAP-16 and *Ophiocordyceps australis s.l*. strain MAP-64 were isolated from an infected *Camponotus* and Ponerine worker ant respectively, collected in Mata do Paraíso forest reserve, Minas Gerais, Brazil. *Ophiocordyceps australis s.l*. strain 1348a and *Ophiocordyceps subramanianii s.l*. strain 1346 were isolated from Ponerine worker ants collected in Kukurantumi fetish forest in the Eastern Region of Ghana. Previously reported *Ophiocordyceps unilateralis s.l*. strain SC16a, collected in South Carolina, USA^17,18^, was also used in this study. This species has recently been described and named *Ophiocordyceps kimflemingiae* ^20^. Fungal cultures were kept in Grace’s insect media (Sigma) and supplemented with 10% (v/v) fetal bovine serum (Gibco). Mycelium for DNA and RNA isolation was grown in 100 mL flasks holding 40 mL media, shaken at 60 rpm at 28 °C.

### DNA isolation, genome sequencing and assembly

Fungal mycelium was harvested by decanting liquid cultures over a Buchner funnel containing Whatmann filter paper. Tissue paper and pressure were applied to drain the mycelium from as much liquid as possible. Dried mycelium was snap frozen in liquid nitrogen and stored at −80 °C upon genomic DNA isolation. About 1 cm^2^ of the frozen mycelium was transferred to liquid nitrogen cooled 2 mL Eppendorf tubes (Greiner) with two metal beads (ø 4.76 mm) each. A Tissuelyser II (Qiagen) with a chilled adapter set (Qiagen) at 24 freq/s for 60 s was used to disrupt fungal cells. To extract DNA, 0.9 mL Extraction Buffer (1% SDS, 24 g/L PAS, 1x RNB (5x RNB: 12.11 g Tris/HCl, 7.304 g NaCl 9.51 EGTA in 100 mL, pH 8.5)) and 0.9 mL phenol/chloroform were used. After spinning down for 10 min at 10.000 rpm, the water phase was transferred to a new tube. Samples were purified using DNA Cleanup columns (Qiagen) and the manufacturer’s protocols. Following elution, samples were RNAse treated with RNAse mix (Ambion) for 10 min at 37 °C. Quality and quantity of the samples were checked on a Bioanalyzer System with the High Sensitivity DNA Analysis Kit (Agilent Technologies) and a Qubit with the DNA BR Assay Kit (Invitrogen).

In an attempt to avoid fragmentation bias, two genomic libraries were prepared for genome sequencing. Nextera libraries (Illumina) were prepared according to manufacturer’s instructions. Additionally, the NEBNext DNA Library Prep Master Mix Set for Illumina (New England Biolabs) was used. To this end, we sheared 5 µg genomic DNA of each sample to an approximate length of 800 bp with a Covaris M220 ultrasonicator with 50 µl screw caps. Fragments of 650–850 bp were selected with a BluePippin electrophoresis unit (Sage Science). A MiSeq sequencer (Illumina) was used to perform 2 × 300 bp paired-end sequencing.

Sequences were trimmed and assembled with CLC Genomics Workbench v7.5 (Qiagen) and default parameters. Not all libraries resulted in high-quality sequencing data. Assembly attempts integrating this lower quality data resulted in more fragmented assemblies. To improve our assemblies, we thus discarded sequencing runs that resulted in lesser quality reads. As such, *O. camponoti-rufipedis* from Brazil, strain Map-64 (*O. australis s.l*. from Brazil) and strain 1348a (*O. australis s.l*. from Ghana) were assembled using only sequenced Nextera libraries. For *O. camponoti-rufipedis* 93.8% of primary sequences assembled into 2206 scaffolds (minimum length 1000 bp, average coverage 29-fold) with a total genome size of 21.91 Mb. For Map-64, 98.2% of primary sequences assembled into 594 scaffolds (minimum length 1000 bp, average coverage 135-fold) with a total genome size of 23.32 Mb. For 1348a, 95.1% of primary sequences assembled into 2297 scaffolds (minimum length 1000 bp, average coverage 20-fold) with a total genome size of 22.19 Mb. Strain 1346 (*O. subramanianii s.l*. from Ghana) was assembled using only sequenced NEBNext libraries. This resulted in 96.0% of primary sequences assembling into 3401 scaffolds (minimum length 1000 bp, average coverage 19-fold) with a total genome size of 32.31 Mb. Previously reported strain SC16a (*O. unilateralis s.l*. from the USA, renamed *O. kimflemingiae*) was also reassembled. For this strain sequences from NEBNext and Nextera libraries could still be combined. This resulted in 93.5% of primary sequences assembling into 2537 scaffolds (minimum length 1000 bp, average coverage 130-fold) with a total genome size of 23.92 Mb (Table 1, and Supplementary Table S1). Repetitive sequences in the assembly were masked using RepeatMaker^36^, RepBase library^37^ and RepeatScout^38^.

### RNA isolation, mRNA-Seq library construction and sequencing

We did not have enough mycelium from *O. camponoti-rufipedis* to generate an RNA sample to aid in gene prediction. For the other strains, enough mycelium was harvested so 1 cm^2^ mycelium could be used for RNA isolation. Total RNA was extracted as previously reported^39^. A Bioanalyzer System with the RNA 6000 Nano Kit (Agilent Technologies) and a Qubit with the RNA BR Assay Kit (Invitrogen) were used to verify sample quality and quantity. mRNA-Seq libraries were constructed with a NEBNext Ultra Directional RNA Library Prep Kit for Illumina (New England BioLabs). Quality and quantity of the resulting cDNA libraries were verified with a Qubit using the ds DNA HS Assay Kit (Invitrogen) and a Bioanalyzer System with the High Sensitivity DNA Analysis Kit (Agilent Technologies). Samples were sequenced on a HiSeq. 1500 (Illumina) using 100 bp paired-end sequencing in rapid-run mode.

### Gene prediction

Genes were predicted for all *Ophiocordyceps* strains in this study, including the reassembled version of previously reported *O. kimflemingiae* strain SC16a^18^. To this end, Augustus version 3.0.2^40^ was trained using BRAKER1 version 1.1.8^41^. To aid in gene prediction, for all strains in this study (except *O. camponoti-rufipedis*), intron hints for Augustus were produced from Tophat transcript alignments using the Augustus software package. Manual inspection of the predicted genes revealed that neighboring genes were occasionally merged into one gene model, separated by a relatively large intron (data not shown). Therefore, a second round of gene prediction was performed. Here, the midpoints of introns larger than 150 bp and not supported by RNA-Seq alignments were labeled as ‘intergenic regions’ in the Augustus hints file. In the resulting gene predictions, these merged genes were usually correctly split into two genes. Completeness of the set of predicted proteins was analyzed using the CEGMA protein set^42^ using a BLASTp E-value cutoff of 1e-5, as well as BUSCO version 2^43^ using the reference dataset ‘pezizomycotina_odb9’ (Table 1).

No RNA-Seq reads were available for *O. camponoti-rufipedis* to aid in gene prediction. To demonstrate that SC16a RNA-Seq reads could not be used directly to inform the gene prediction for this species, even though both species reside within the same species complex (*O. unilateralis s.l*.) reads were also mapped across genomes. This was done with CLC Genomics Workbench v7.5 (Qiagen) using default parameters. In this comparison, the assembled genome of *Ophiocordyceps polyrhachis* ^22^ was also included (this genome could however not be used in the comparative analyses below since its annotation has not been publically deposited). Instead of using the SC16a transcripts directly to predict genes in *O. camponoti-rufipedis*, we chose an approach that used them indirectly. Augustus version 3.0.2^40^ was used to predict genes, using the RNA-Seq-informed parameter files previously generated by BRAKER1 version 1.1.8^41^. for *O. kimflemingiae* SC16a (described above).

### Functional annotation

We functionally annotated the predicted proteins of all *Ophiocordyceps* strains in this study. PFAM version 27^44^ was used to predict conserved protein domains, which were subsequently mapped to the corresponding gene ontology (GO) terms^45,46^. The MEROPS database^47^ with a BLASTp E-value cutoff of 1e-5 was used to predict proteases. Secretion signals were predicted with Signalp 4.1^48^. Transmembrane domains were predicted with TMHMM 2.0c^49^. Proteins were considered small secreted proteins when they had a secretion signal, but no transmembrane domain (except in the first 40 amino acids) and were shorter than 300 amino acids. A pipeline based on the SMURF method^50^ was used to predict genes and gene clusters involved in secondary metabolism. SMURF parameter d (maximum intergenic distance in base pairs) was set at 3000 bp. SMURF parameter y (the maximum number of non-secondary metabolism genes upstream or downstream of the backbone gene) was set at 6.

In cases where additional homology searches were performed against the NCBI database, Blast2Go^51^ with BLASTp default settings was used.

### Comparative analyses

For comparative purposes, 18 previously published genomes of fungal acomycetes were included that represent various fungal life styles within and outside the Order Hypocreales: *Ophiocordyceps sinensis* ^52^, *Ophiocordyceps minnesotensis* ^53^, *Drechmeria coniospora* ^33^, *Tolypocladium inflatum* ^54^, *Cordyceps* militaris^55^, *Cordyceps bassiana* ^34^, *Metarhizium robertsii* and *Metarhizium acridum* ^32^, *Claviceps purpurea* ^56^, *Fusarium oxysporum* ^57^, *Fusarium graminearum* ^58^, *Trichoderma reesei* ^59^, *Trichoderma virens* ^60^, *Magnaporthe oryzae* ^31^, *Candida albicans* ^61^, *Aspergillus fumigatus* ^62^, *Neurospora crassa* ^63^, *Aspergillus nidulans* ^64^. To estimate the phylogeny of these organisms, 67 conserved genes that were found present in each organism were aligned using MAFFT version 7.123b^65^. Well-aligned regions were identified using Gblocks 0.91b^66^. This resulted in 41793 amino acid positions. The parallelized version of RAxML version 8.1.16^67^ with the PROTGAMMAWAG model with 100 rapid bootstrap partitions was used to reconstruct a species tree. The tree was visualized using iTOL^68^.

Proteins that contained a ‘heat-labile enterotoxin alpha chain’ domain (PFAM domain PF01375) were identified as enterotoxins. The domains were aligned using MAFFT version 7.123b^65^. Next, a phylogenetic gene tree was reconstructed with FastTree 2.0 using default parameters^69^. The tree was visualized using iTOL^68^, and rooted on the heat-labile enterotoxin alpha chain’ domain of bacterium *Leptospira mayottensis*.

Clusters of orthologous genes among the five *Ophiocordyceps* genomes produced in this study, as well as the comparative genomes, were identified using OrthoMCL version 2.0.9^70^ with an all-versus-all blastp E-value cutoff of 1e-5 and an inflation factor of 1.5. Venn diagrams demonstrating ortholog cluster commonalities between species (ranges) were visualized with http://bioinformatics.psb.ugent.be/webtools/Venn. Over- and under-representation of functional annotation terms were calculated using the Fisher Exact test with the Benjamini-Hochberg correction to correct for multiple testing (corrected *p*-value < 0.05).

### Transcriptomics analysis

Previously published mixed transcriptomics data was used again in this study to further investigate fungal gene expression during and after manipulated biting behavior^18^. Sequence tags were mapped to the new genome version of strain SC16a (*O. unilateralis s.l*. from the USA) and differentially expressed genes were identified using the methods previously described^18^. Genes found to be significantly up-regulated during manipulated biting behavior followed by a significant down-regulation after were subjected to further comparative analyses.

### Data availability

The Whole Genome Shotgun projects in this study have been deposited at DDBJ/EMBL/GenBank under the accession numbers LAZP02000000 (bio sample number SAMN03465111) and SUB2752196, (bio sample numbers SAMN07142922, SAMN07142923, SAMN07142924, SAMN07142925). Genome information can also be accessed through http://fungalgenomics.science.uu.nl/. The RNA-Seq data generated in this study is available through NCBI’s Sequence Read Archive under the biosample accessions SAMN07193086, SAMN07193087, SAMN07193088. All other data generated or analyzed during this study are included in this published article and its Supplementary Data files.

## Electronic supplementary material

Dataset 1

Supplementary Files
