# Supplementary material for: Ant-infecting Ophiocordyceps genomes reveal a high diversity of potential behavioral manipulation genes and a possible major role for enterotoxins
Source: Sci Rep. 2017 Oct 2;7:12508. doi: 10.1038/s41598-017-12863-w (PMC5624889; doi:10.1038/s41598-017-12863-w)
Supplement: Supplementary file 2 — Supplementary Files [file 41598_2017_12863_MOESM2_ESM.pdf]

Ant-infecting *Ophiocordyceps* genomes reveal a high diversity of potential behavioral manipulation genes and a possible major role for enterotoxins

Charissa de Bekker[1,2,3*], Robin A Ohm[4], Harry C Evans[5], Andreas Brachmann[2], and David P Hughes[6*]

[1]University of Central Florida, Department of Biology, Orlando, FL 32816, USA
[2]Ludwig Maximilian University, Faculty of Biology, Planegg-Martinsried, 82152, Germany
[3]Ludwig Maximilian University, Institute of Medical Psychology, Munich, 80336, Germany
[4]Utrecht University, Faculty of Science, Utrecht, 3584 CH, The Netherlands
[5]CAB International, E-UK, Egham, Surrey TW20 9TY, United Kingdom
[6]Pennsylvania State University, Entomology and Biology Departments, University Park, PA 16802, USA
[*]charissa.debekker@ucf.edu; dph14@psu.edu

Supplementary Table S1 *Ophiocordyceps kimflemingiae* (previously *O. unilateralis s.l.* strain SC16a) genome assembly and annotation statistics

**Property**	***O. kimflemingiae*** **Version 1 (de Bekker et al., 2015)**	***O. kimflemingiae*** **Version 5 (this study)**
Fold coverage	120	130
Scaffolds in assembly	7875	2537
Total assembly length (Mbp)	26.05	23.92
N50 (kb)	22	27
Largest scaffold (kb)	145	167
Assembly GC content (%)	54.76	55.92
Assembly gaps (%)	0.73	0.75
Repetitive content (%)	7.81	6.83
Genes	7831	8629
Gene length (median)	1420	1294
Transcript length (median)	1275	1152
Exon length (median)	261	220
CDS length (median)	1272	1149
Protein length (median)	424	383
Spliced genes (total, %)	6038 (77.1%)	6871 (79.63%)
Exons per gene (median)	3	3
Intron length (median)	62	62
Introns per spliced gene (median)	3	2
Gene density (genes / Mbp)	300.57	360.69
Unique PFAM domains	3498	3499
Genes with PFAM (total, %)	5556 (70.95%)	5749 (66.62%)
Genes with GO (total, %)	3904 (49.85%)	3981 (46.14%)
Genes with signalP (total, %)	891 (11.38%)	914 (10.59%)
Genes with TMHMM (total, %)	1493 (19.07%)	1536 (17.8%)
CEGMA completeness (%)	98.69	99.13
BUSCO complete or fragmented (%)	96.5	97.6

Supplementary Table S2 Reads mapping across genomes. Percentage of RNA-Seq reads, generated from four different *Ophiocordyceps* species, mapped to the genome assembly of the same *Ophiocordyceps* species (in bold), species within the same complex (underlined) and outside the species complex.

**Assemblies**	**% Mapped reads**<br>*O. kimflemingiae*<br>- USA	*O. australis s.l.*<br>- Brazil	*O. australis s.l.*<br>– Ghana	*O. subramanianii s.l.*<br>- *Ghana*
O. kimflemingiae<br>– USA (SC16a)	**93.41**	32.26	31.79	48.3
O. camponoti-rufipedis<br>- Brazil (Map-16)	<u>42.63</u>	32.9	32.83	48.82
O. polyrhachis-furcata<br>- Thailand (LKCN01)	<u>40.52</u>	31.17	31.18	46.32
O. australis s.l.<br>- Brazil (Map-64)	14.48	**86.4**	<u>82.17</u>	37.19
O. australis s.l.<br>– Ghana *(1348a)*	14.21	<u>70.6</u>	**96.55**	37.18
O. subramanianii s.l.<br>- Ghana *(1346)*	28.21	34.11	34.8	**98.02**

Supplementary Table S3 Blast results of ant infection-specific orthologous clusters shared among all five *Ophiocordyceps* genomes. BLASTp results of all genes residing within the four clusters that were found to be specific for ant-infecting fungi, and present in all five species studied here.

ProteinID	SignalP	Description	Length	#Hits	e-Value	sim mean
**ClusterID 8742**						
Ophio5|3517	no	hypothetical protein XA68_3158 *O. unilateralis s.l.*	430	1	0.00E+00	100%
Ophun1|2841	no	hypothetical protein XA68_3158 *O. unilateralis s.l.*	633	1	2.63E-90	63%
OphauB2|340	no	hypothetical protein XA68_3158 *O. unilateralis s.l.*	607	1	7.82E-18	45%
OphauB2|718	no	hypothetical protein XA68_3158 *O. unilateralis s.l.*	681	1	2.40E-18	46%
OphauB2|992	no	hypothetical protein XA68_3158 *O. unilateralis s.l.*	680	1	2.10E-06	59%
OphauG2|2031	no	hypothetical protein XA68_3158 *O. unilateralis s.l.*	610	1	1.98E-15	54%
Hirsu2|6266	no	---NA---	142			
**ClusterID 8743**						
Ophio5|3518	yes	metalloprotease partial *M. brunneum* ARSEF 3297	701	14	0.00E+00	45.29%
Ophun1|2840	yes	metalloprotease 1 *M. rileyi* RCEF 4871	574	7	0.00E+00	46.14%
OphauB2|5232	no	metalloprotease MEP1, partial *M. majus* ARSEF 297	380	20	2.21E-45	43.50%
OphauB2|993	no	Extracellular metalloprotease *C. higginsianum* IMI 349063	522	20	3.09E-100	48.40%
OphauG2|1200	yes	metalloprotease 1, partial *M. majus* ARSEF 297	657	20	5.77E-79	47.95%
OphauG2|7035	yes	metalloprotease 1, partial *M. majus* ARSEF297	677	20	3.59E-81	45.85%
Hirsu2|655	yes	metalloprotease, *M. bolleyi*	699	20	1.63E-121	47.10%
**ClusterID 9202**						
Ophio5|3293	yes	hypothetical protein XA68_2954 *O. unilateralis s.l.*	389	6	0.00E+00	52%
Ophun1|3187	yes	hypothetical protein XA68_2954 *O. unilateralis s.l.*	389	7	1.24E-174	47.57%
OphauB2|1560	yes	hypothetical protein XA68_2954 *O. unilateralis s.l.*	298	4	9.70E-30	44.25%
OphauB2|341	no	hypothetical protein XA68_2954 *O. unilateralis s.l.*	244	7	7.19E-29	41.57%
OphauG2|1201	no	hypothetical protein XA68_2954 *O. unilateralis s.l.*	256	4	2.07E-26	42.75%
Hirsu2|656	yes	hypothetical protein XA68_2954 *O. unilateralis s.l.*	305	6	1.02E-23	41.17%
**ClusterID 9833**						
Ophio5|6487	no	hypothetical protein XA68_533 *O. unilateralis s.l.*	366	4	0.00E+00	56.75%
Ophun1|527	no	hypothetical protein XA68_533 *O. unilateralis s.l.*	367	5	1.26E-95	47.60%
OphauB2|4854	yes	hypothetical protein XA68_533 *O. unilateralis s.l.*	366	6	9.34E-77	44.33%
OphauG2|783	yes	hypothetical protein XA68_533 *O. unilateralis s.l.*	368	4	3.94E-72	47.25%
Hirsu2|4982	no	hypothetical protein XA68_533 *O. unilateralis s.l.*	315	4	2.56E-58	43.75%

Supplementary Table S4 Blast results of manipulation-specific orthologous clusters shared among all four *Ophiocordyceps* species that induce biting behavior. BLASTp results of all genes residing within the four clusters that were found to be specific for fungi that induce biting behavior, and present in all four species studied here.

ProteinID	SignalP	Description	Length	#Hits	e-Value	Identity
**ClusterID 10685**						
Ophio5|3593	no	hypothetical protein XA68_1158 *O. unilateralis s.l.*	214	6	2E-158	100%
Ophun1|1827	no	hypothetical protein XA68_1158 *O. unilateralis s.l.*	216	6	2E-109	70%
OphauG2|4155	yes	hypothetical protein XA68_1158 *O. unilateralis s.l.*	270	9	2E-52	41%
Hirsu2|7386	no	hypothetical protein XA68_1158 *O. unilateralis s.l.*	269	5	2E-20	29%
**ClusterID 10761**						
Ophio5|7380	yes	hypothetical protein XA68_6681 *O. unilateralis s.l.*	804	4	0E+00	100%
Ophun1|2688	no	hypothetical protein XA68_6681 *O. unilateralis s.l.*	789	6	0E+00	67%
OphauG2|4227	yes	hypothetical protein XA68_6681 *O. unilateralis s.l.*	808	3	5 E-91	30%
Hirsu2|10631	yes	hypothetical protein XA68_6681 *O. unilateralis s.l.*	827	10	5E-121	37%

Supplementary Figure S1 Orthologous clustering summary statistics of all ascomycetes in this study

Each species was found to have their predicted proteins dispersed across 12.9 % to 27.7 % of the total amount of clusters (A). This percentage appeared to be positively correlated with the amount of predicted proteins per species genome (B). The manipulating *Ophiocordyceps* species followed this trend with the species with smaller proteomes (*O. unilateralis s.l.* and *O. australis s.l.*) having proteins across 14.4-16.2 % of the clusters, and the bigger *O. subramanianii* proteome having proteins across 20.8 % of the clusters. *C. albicans*, *C. purpurea* and *O. minnesotensis* were outliers (B). Generally, >90 % of all predicted proteins within a species genome were uniquely allocated to one of the PFAM clusters (C). Only five species dropped below this percentage, including *C. albicans*, which with ~50% of the proteome having two or more proteins per orthologous cluster represents a diploid genome. The manipulating *Ophiocordyceps* species generally had the most single proteins per PFAM cluster with 95.3-96.3 % of all proteins for *O. unilateralis s.l.* and *O. australis s.l.*, and 93.9 % of all *O. subramarianii s.l.* proteins (C). The percentage of proteins with a unique PFAM cluster did not appear to be correlated to species proteome size (D).

a

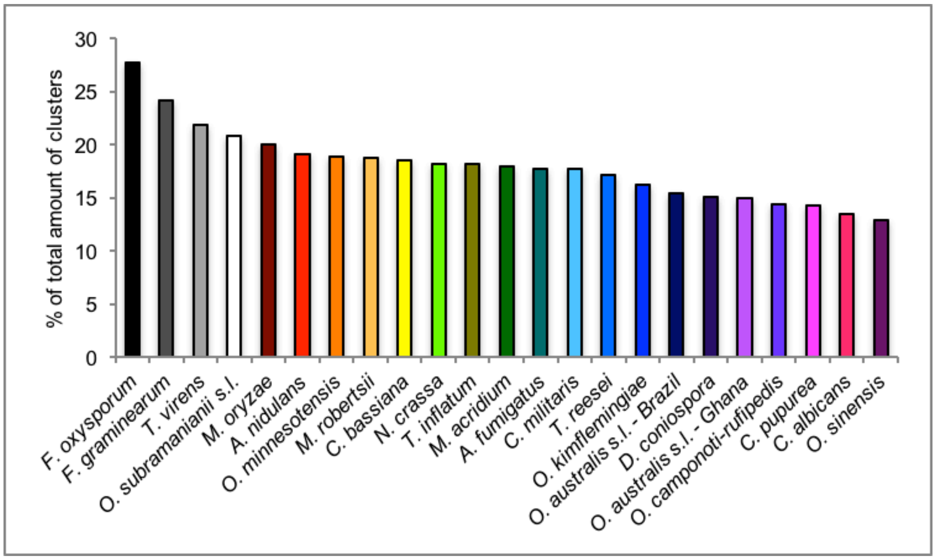

b

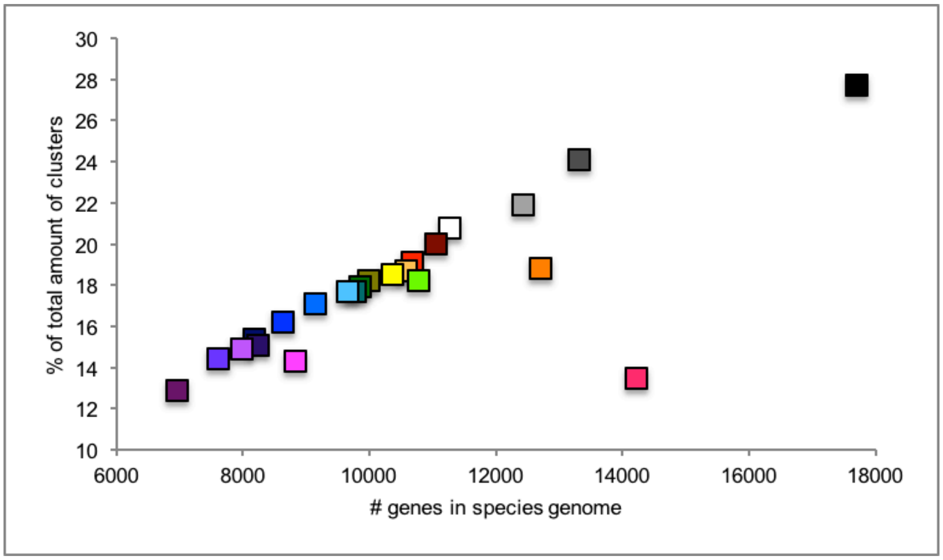

c

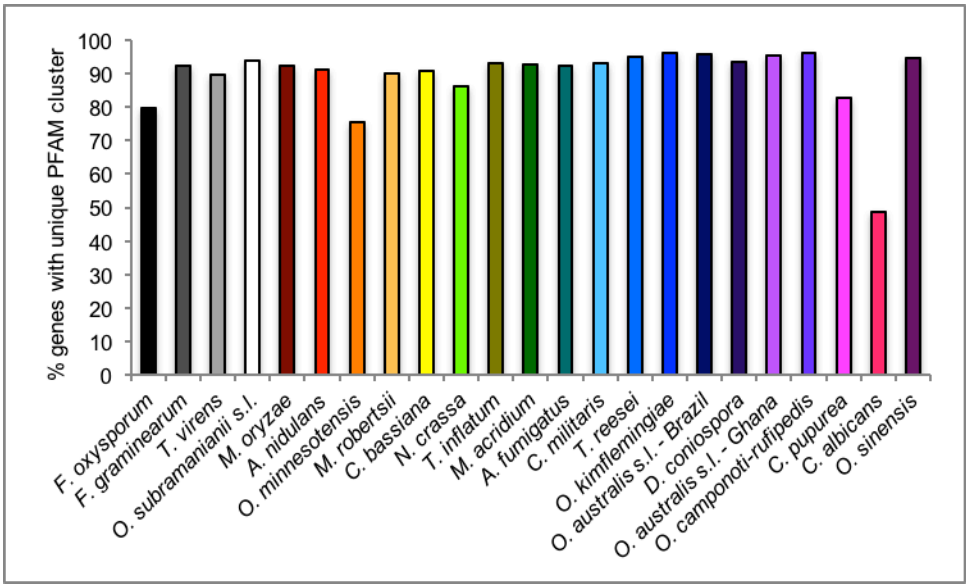

% genes with unique PFAM cluster
100
90
80
70
60
50
40
30
20
10
0
F. oxysporum
F. graminearum
T. virens
O. subramanianii s.l.
M. oryzae
A. nidulans
O. minnesotensis
M. robertsii
C. bassiana
N. crassa
T. inflatum
M. acridium
A. fumigatus
C. militaris
T. reesei
O. kimflemingiae
O. australis s.l. - Brazil
D. coniospora
O. australis s.l. - Ghana
O. camponoti-rufipedis
C. pupurea
C. albicans
O. sinensis

d

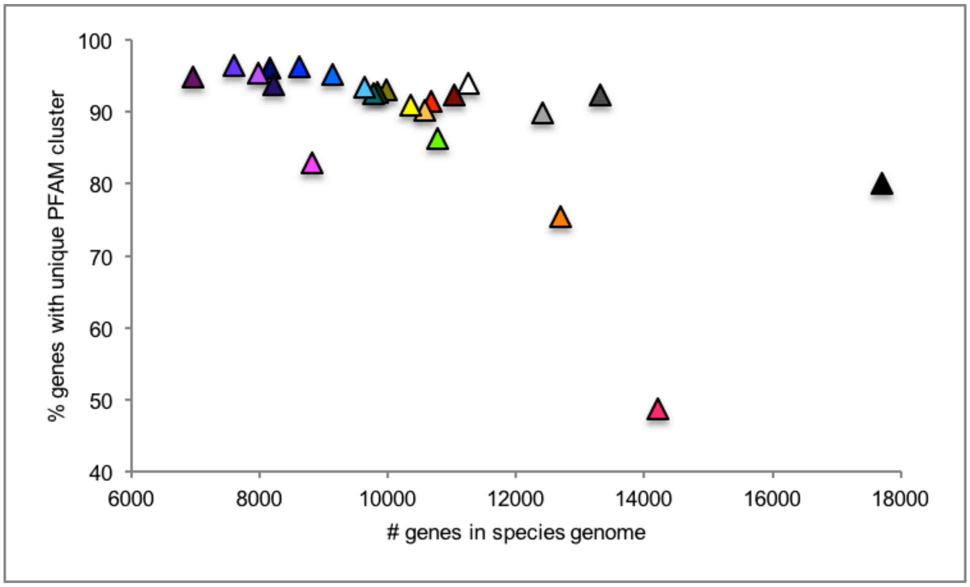

% genes with unique PFAM cluster
100
90
80
70
60
50
40
6000
8000
10000
12000
14000
16000
18000
genes in species genome

Supplementary Figure S2. A Venn-diagram of all orthologous clusters found in ant-manipulating species.

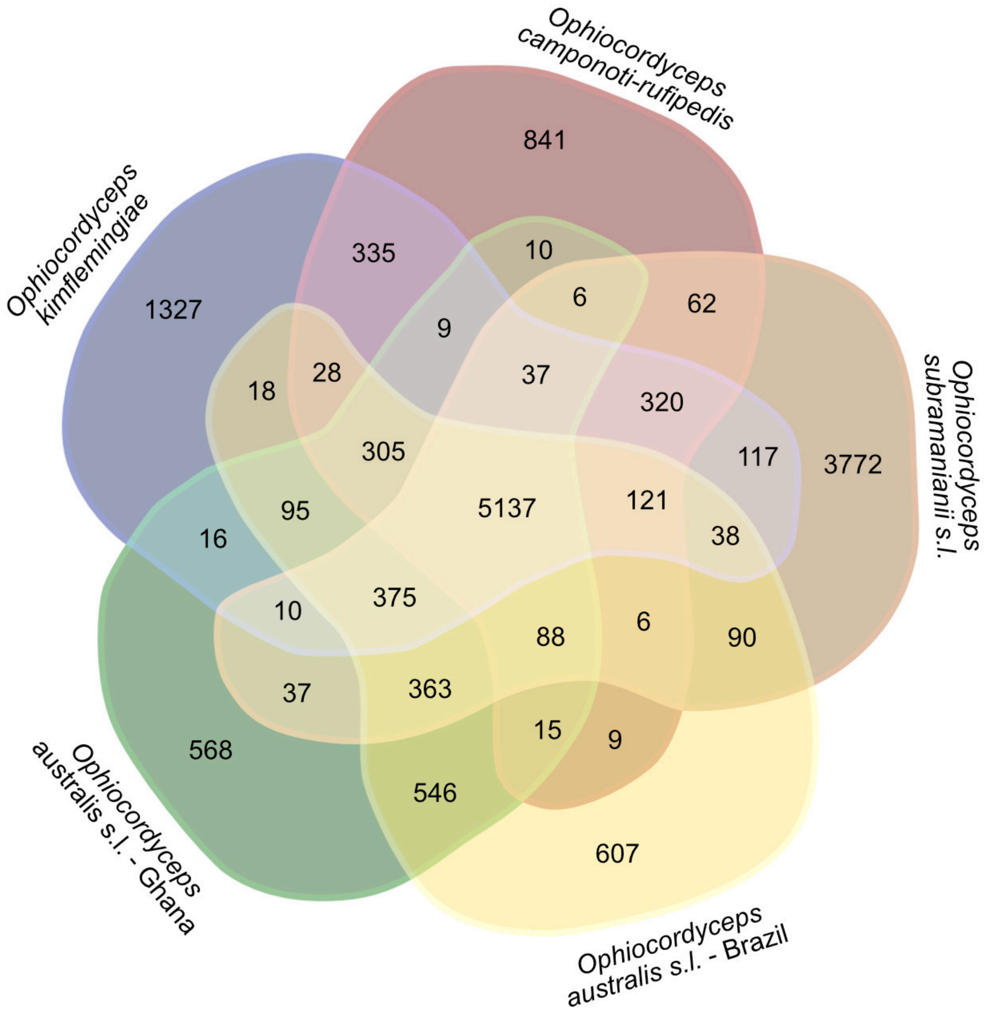

Supplementary Figure S3: Secondary metabolite clusters in ant-infecting *Ophiocordyceps* species. Homology (identified by BLASTp alignment) and orthology (identified by orthologous clustering) of genes within A) secondary metabolite cluster 7 and B) secondary metabolite cluster 9. Genes that were found through both methods are connected by a black line. Genes found by BLASTp alignment only are connected with a blue line, while genes only found by orthologous clustering are connected with a dashed line. Gene numbers and functions in red indicate genes that received a secondary metabolism annotation.

a

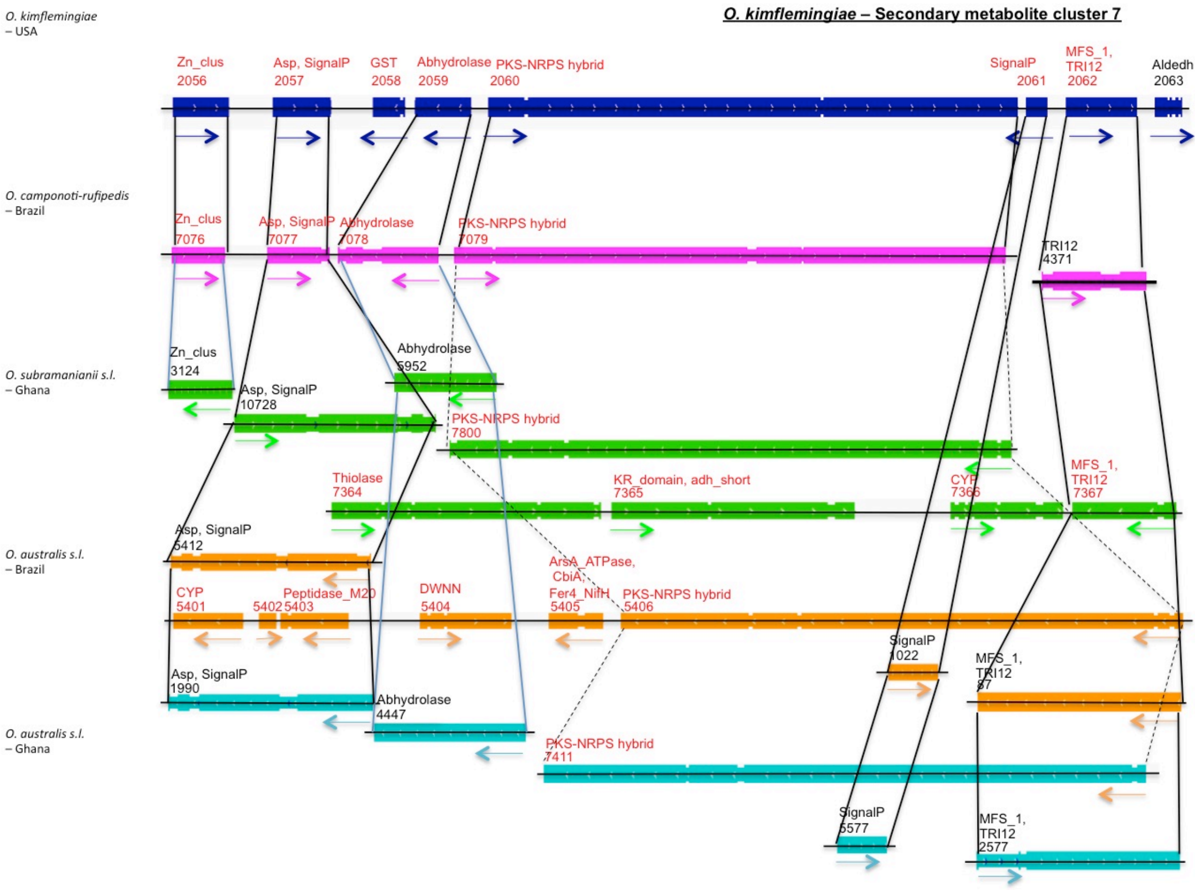

b

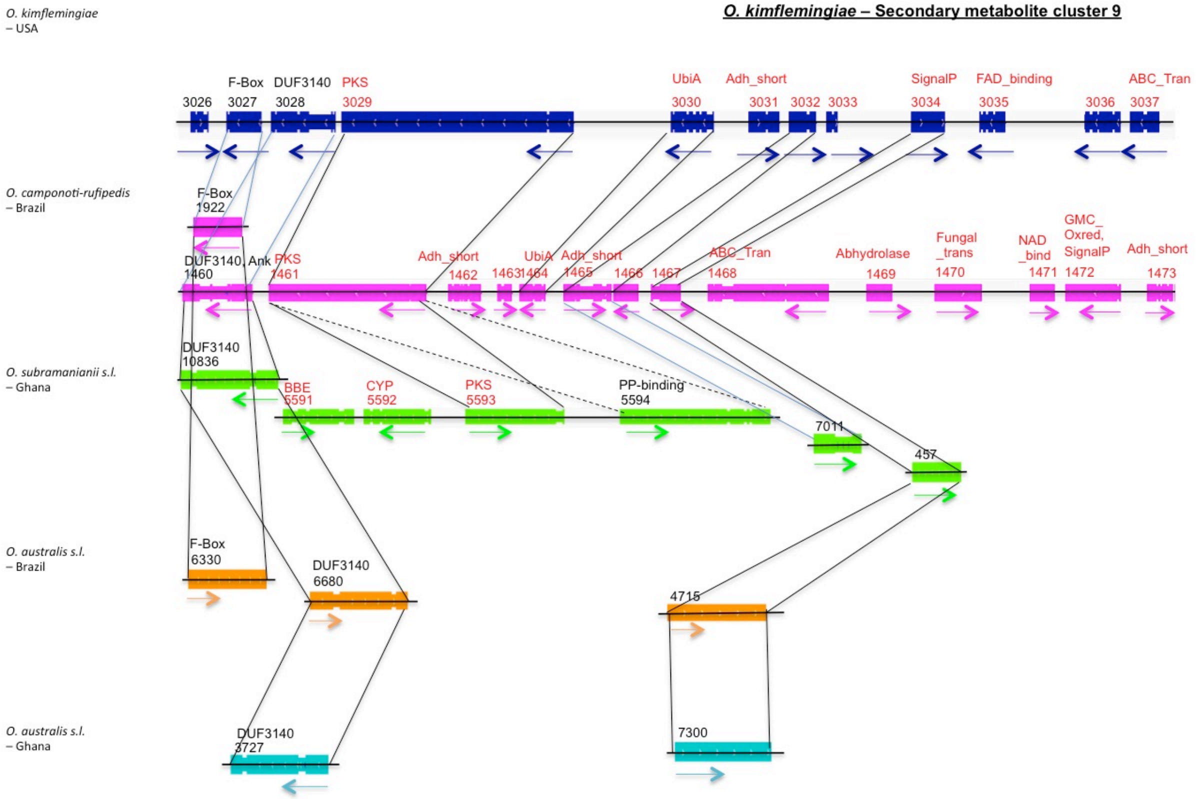

Supplementary Figure S4 Phylogenetic relationship of putative enterotoxins. This tree is based on the functional domains (PF01375) of all putative enterotoxins found across the fungal species compared in this study and in the bacterial species *L. mayottensis*, *V. cholerae*, and *E. coli* (ETEC 1392/75 and H10407). Species ranges are indicated with different colors. Ant-infecting *Ophiocordyceps* species are indicated with different shades of red. *Ophiocordyceps kimflemingiae* enterotoxins that showed differential expression patterns during and after manipulated biting behavior are additionally marked with # or *.

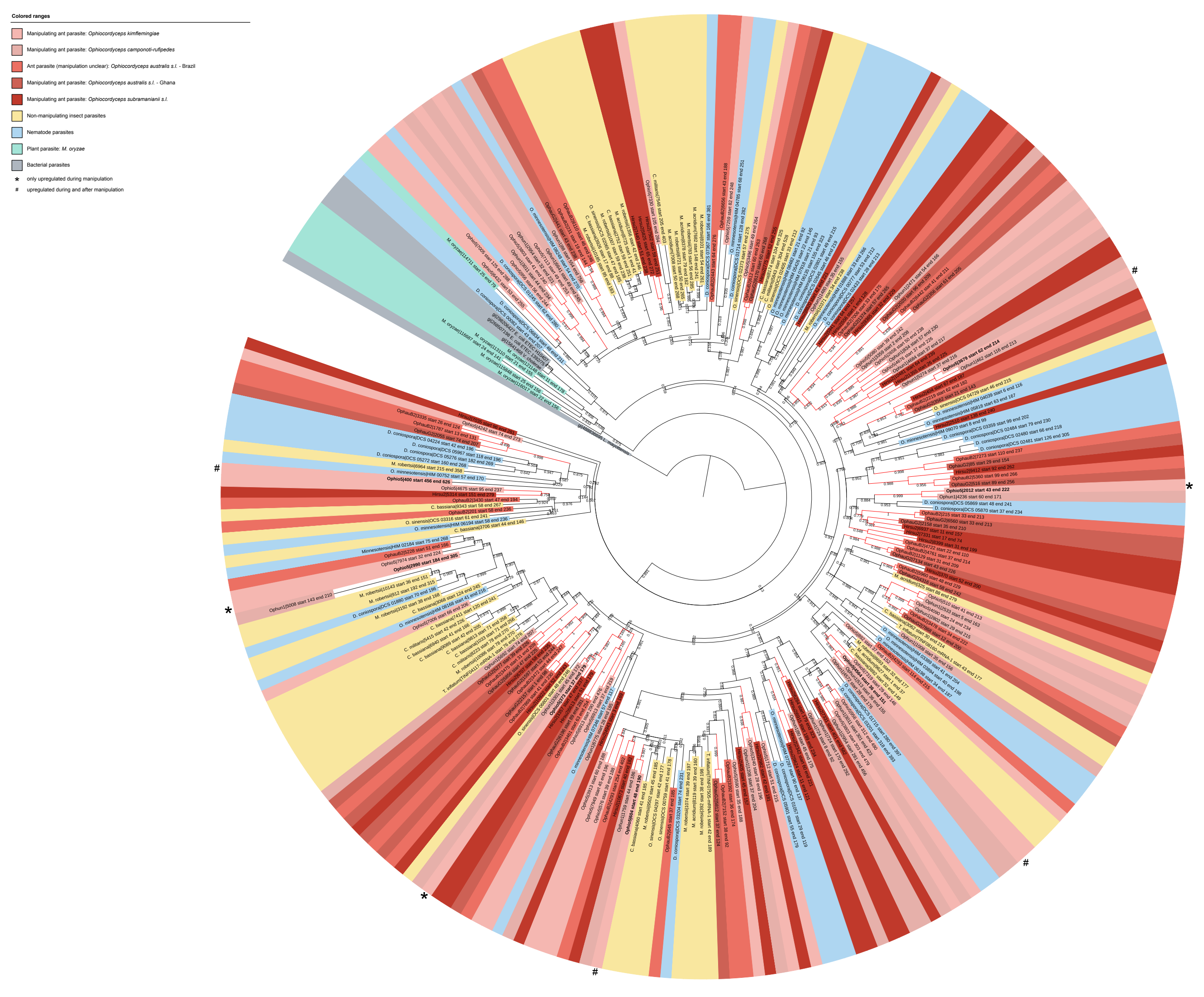

Colored ranges
Manipulating ant parasite: *Ophiocordyceps kimflemingiae*
Manipulating ant parasite: *Ophiocordyceps camponoti-rufipedes*
Ant parasite (manipulation unclear): *Ophiocordyceps australis s.l.* - Brazil
Manipulating ant parasite: *Ophiocordyceps australis s.l.* - Ghana
Manipulating ant parasite: *Ophiocordyceps subramaniani s.l.*
Non-manipulating insect parasites
Nematode parasites
Plant parasite: *M. oryzae*
Bacterial parasites
* only upregulated during manipulation
# upregulated during and after manipulation
